# Deep learning for detection of radiographic sacroiliitis: achieving expert-level performance

**DOI:** 10.1186/s13075-021-02484-0

**Published:** 2021-04-08

**Authors:** Keno K. Bressem, Janis L. Vahldiek, Lisa Adams, Stefan Markus Niehues, Hildrun Haibel, Valeria Rios Rodriguez, Murat Torgutalp, Mikhail Protopopov, Fabian Proft, Judith Rademacher, Joachim Sieper, Martin Rudwaleit, Bernd Hamm, Marcus R. Makowski, Kay-Geert Hermann, Denis Poddubnyy

**Affiliations:** 1 Department of Radiology, Charité — Universitätsmedizin Berlin, Hindenburgdamm 30, 12203 Berlin, Germany; 2 Berlin Institute of Health, BIH, Berlin, Germany; 3 Department of Gastroenterology, Infectious Diseases and Rheumatology, Charité — Universitätsmedizin Berlin, Berlin, Germany; 4 Department of Internal Medicine and Rheumatology, Klinikum Bielefeld Rosenhöhe, Bielefeld, Germany; 5 Department of Diagnostic and Interventional Radiology, School of Medicine, Technical University of Munich, Munich, Germany; 6 Department of Epidemiology, German Rheumatism Research Centre, Berlin, Germany

**Keywords:** Axial spondyloarthritis, Sacroiliitis, Artificial intelligence, Deep learning, Machine learning

## Abstract

**Background:**

Radiographs of the sacroiliac joints are commonly used for the diagnosis and classification of axial spondyloarthritis. The aim of this study was to develop and validate an artificial neural network for the detection of definite radiographic sacroiliitis as a manifestation of axial spondyloarthritis (axSpA).

**Methods:**

Conventional radiographs of the sacroiliac joints obtained in two independent studies of patients with axSpA were used. The first cohort comprised 1553 radiographs and was split into training (*n* = 1324) and validation (*n* = 229) sets. The second cohort comprised 458 radiographs and was used as an independent test dataset. All radiographs were assessed in a central reading session, and the final decision on the presence or absence of definite radiographic sacroiliitis was used as a reference. The performance of the neural network was evaluated by calculating areas under the receiver operating characteristic curves (AUCs) as well as sensitivity and specificity. Cohen’s kappa and the absolute agreement were used to assess the agreement between the neural network and the human readers.

**Results:**

The neural network achieved an excellent performance in the detection of definite radiographic sacroiliitis with an AUC of 0.97 and 0.94 for the validation and test datasets, respectively. Sensitivity and specificity for the cut-off weighting both measurements equally were 88% and 95% for the validation and 92% and 81% for the test set. The Cohen’s kappa between the neural network and the reference judgements were 0.79 and 0.72 for the validation and test sets with an absolute agreement of 90% and 88%, respectively.

**Conclusion:**

Deep artificial neural networks enable the accurate detection of definite radiographic sacroiliitis relevant for the diagnosis and classification of axSpA.

## Background

Axial spondyloarthritis (axSpA) is a chronic inflammatory disease that mainly affects the axial skeleton, the sacroiliac joints and the spine. For many years, the detection of radiographic sacroiliitis has been the only way to make a definite diagnosis of the disease prior to the development of structural spinal damage. The presence of definite radiographic sacroiliitis (defined as sacroiliitis of at least grade 2 bilaterally or at least grade 3 unilaterally) is also a mandatory criterion of the modified New York criteria for ankylosing spondylitis (AS) [1]. Although magnetic resonance imaging (MRI) of the sacroiliac joints nowadays enables earlier diagnosis of axSpA, definite radiographic sacroiliitis can be detected at the time of diagnosis in about 33% of the patients with symptoms lasting up to 1 year and in about 50% of the patients with a symptom duration of 2 to 3 years [2]. Conventional radiography of the sacroiliac joints is therefore still recommended as the first imaging method in patients with suspected axSpA [3]. Furthermore, radiographic sacroiliitis — together with sacroiliitis on MRI — is included in the Assessment of Spondyloarthritis International Society (ASAS) classification criteria for axSpA [4]. Depending on the presence or absence of definite radiographic sacroiliitis, axSpA can be classified as either radiographic axSpA (r-axSpA, synonymous to AS) or non-radiographic axSpA (nr-axSpA) [5]. Such a classification could be relevant for both clinical practice (currently, the labels for biological disease-modifying antirheumatic drugs — bDMARDs are different for AS and nr-axSpA) and research (i.e., stratification or selection of patients in a clinical trial).

Although conventional radiography of the sacroiliac joints still plays an important role in both clinical practice and clinical trials, its reliability has been reported as mostly poor in a number of studies, even when assessed by expert readers [6–10]. In addition, it has been shown that untrained local readers perform worse than expert readers specialised in SpA [10]. One possible solution to achieve a comparable high accuracy as an expert in detecting radiographic sacroiliitis, even in non-specialised clinics, could be to develop an artificial intelligence-based model for the analysis of radiographs.

Deep learning has already produced remarkable results in the classification of medical and non-medical data. For example, deep neural networks have been trained to detect breast cancer in mammographs, to classify skin cancer or to label chest radiographs [11–13]. In all of these studies, the investigators did not develop a de novo model but applied a transfer learning approach using a pre-trained network. Such an approach allows the knowledge of pre-trained models from non-medical fields to be used for a new visual task, effectively reducing the amount of data required for training while increasing the accuracy of the models.

In the present study, we therefore aimed to develop and validate a deep neural network for the detection of definite radiographic sacroiliitis, using centrally scored images from two observational cohort studies.

## Methods

### Cohort description

In this project, we used imaging data from two independent sources: (1) *P*atients With Axial Spondyloa*r*thritis: Multic*o*untry Registry *of* Clinical Characteristics (PROOF) and (2) German Spondyloarthritis Inception Cohort (GESPIC).

PROOF is an ongoing study conducted in clinical practices in 29 countries and includes 2170 adult patients diagnosed with axSpA (non-radiographic or radiographic) ≤ 12 months before study enrolment and fulfilling the ASAS classification criteria for axSpA. In 1553 patients, radiographs of the sacroiliac joints were available for central reading.

GESPIC is a multicentre inception cohort study conducted in Germany and includes 525 patients with axSpA [14]. In 458 patients, radiographs of the sacroiliac joints were available for central reading.

Baseline characteristics of both cohorts are presented in Table 1.

**Table 1:** Baseline characteristics of patients with axial spondyloarthritis from the PROOF and GESPIC cohorts

Parameter at baseline	PROOF (***n*** = 1553)	GESPIC (n = 458)
Age, years, mean (SD)	34.7 (10.5)	35.7 (10.3)
Male sex, *n* (%)	983 (63.3)	243 (53.1)
Duration of symptoms, years, mean (SD)	4.7 (6.8)	4.0 (2.7)
HLA-B27 positive, *n* (%)	836 (64.6)	359 (78.4)
CRP, mg/l, mean (SD)	15.6 (23.0)	11.5 (18.2)
ASDAS-CRP, mean (SD)	2.9 (1.1)	2.6 (1.0)
BASDAI, 0–10, mean (SD)	4.5 (2.3)	3.9 (2.1)
BASFI, 0–10, mean (SD)	3.3 (2.5)	2.8 (2.3)
Peripheral arthritis, *n* (%)	503 (32.4)	65 (14.2)
Uveitis, *n* (%)	151 (9.7)	79 (17.3)
Psoriasis, *n* (%)	106 (6.8)	49 (10.7)
IBD, *n* (%)	40 (2.6)	12 (2.6)
Family history of SpA, *n* (%)	291 (18.7)	147 (32.1)
Treatment with NSAIDs, *n* (%)	1204 (77.5)	305 (66.6)
Treatment with csDMARDs, *n* (%)	539 (34.7)	107 (23.4)
Treatment with systemic steroids, *n* (%)	119 (7.7)	40 (8.7)
Treatment with a TNF inhibitor, n (%)	234 (15.1)	11 (2.4)

*ASDAS-CRP* C-reactive protein-based ankylosing spondylitis disease activity score, *axSpA* axial spondyloarthritis, *BASDAI* Bath Ankylosing Spondylitis Disease Activity Index, *BASFI* Bath Ankylosing Spondylitis Functional Index, *CRP* C-reactive protein, *csDMARDs* conventional synthetic disease-modifying antirheumatic drugs, *IBD* inflammatory bowel disease, *nr-axSpA* non-radiographic axial SpA, *NSAIDs* non-steroidal anti-inflammatory drugs, *r-axSpA* radiographic axial SpA, *SD* standard deviation, *SpA* spondyloarthritis, *TNF* tumour necrosis factor

### Assessment of radiographic sacroiliitis

Radiographs of the sacroiliac joints were collected, digitised if necessary, anonymised and subsequently centrally graded by trained and calibrated readers using the modified New York criteria [1]:

**Table:** 

Grade 0	Normal
Grade 1	Suspicious changes
Grade 2	Minimal abnormality: small localised areas with erosion or sclerosis, without alteration in the joint width
Grade 3	Unequivocal abnormality: moderate or advanced sacroiliitis with erosions, evidence of sclerosis, widening, narrowing or partial ankylosis
Grade 4	Severe abnormality: total ankylosis

In the PROOF study, images were first assessed by the local readers, then by central reader 1 (DP, board-certified rheumatologist with more than 10 years of experience in SpA imaging assessment), who was blinded to the results of the local assessment. In case of a disagreement on the presence of definite radiographic sacroiliitis (grade ≥ 2 bilaterally or grade ≥ 3 unilaterally) between the local and central reader 1, the radiograph was evaluated by central reader 2 (HH, board-certified rheumatologist with more than 10 years of experience in SpA imaging assessment), who was blinded to the previous assessments. The final decision on the presence of definite radiographic sacroiliitis and, therefore, on the classification as nr-axSpA or r-axSpA, was made based on the decision of two of the three readers.

In GESPIC, no local reading of radiographs was demanded; all collected images were scored independently by two trained and calibrated central readers (VRR and MT, board-certified rheumatologists with approximately 5 years of experience in SpA imaging assessment).

### Image selection and pre-processing

**Fig. 1:**
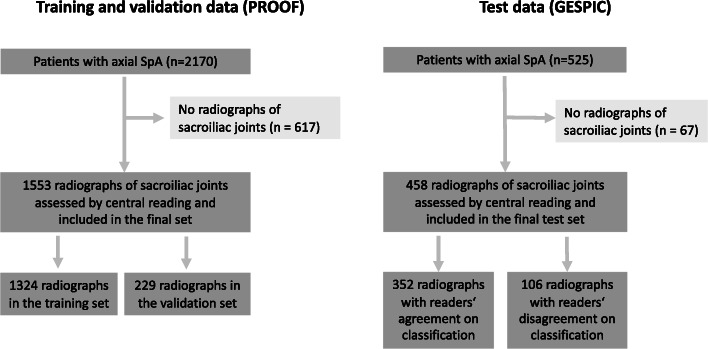
Flowchart for the selection of cases from the PROOF (training and validation set) and GESPIC (test set) studies

The PROOF dataset consists of 1553 radiographs of the sacroiliac joints in DICOM (Digital Imaging and Communications in Medicine) format, varying in size, resolution and quality (Fig. 1). The Horos Project DICOM Viewer (version 4.0.0, www.horosproject.org) was used to adjust the greyscale levels of all images and to convert them to the Tagged Image File Format (TIFF) afterwards. Images including other body parts such as the thoracic spine were manually cropped to the pelvis. The final dataset for building the model was split randomly into training (1324 radiographs, 85%) and validation datasets (229 radiographs, 15%).

For testing the generalisability across datasets, we defined two subgroups in the GESPIC dataset: (1) patients with the presence or absence of definite radiographic sacroiliitis in the opinion of two readers (*n* = 352) and (2) patients with disagreement of the central readers on the final classification (*n* = 106). Images in the test dataset were pre-processed exactly as the training and validation datasets.

### Model training

Model training was performed on a dedicated Ubuntu 18.04 workstation with two Nvidia GeForce RTX 2080ti graphic cards as well as on a GPU node of the Berlin Institute of Health (BIH) high-performance computing cluster using four Nvidia Tesla V100 graphic cards. All model training was mainly performed using Python (version 3.7) including the fastAI application programming interface, which is built on top of PyTorch [15, 16].

As a base model, we used a convolutional neural network (ResNet-50 architecture) pre-trained on the ImageNet-1k dataset, which includes over 1.28 million images [17]. The images were augmented prior to training through various transformations including flipping, rotation of up to 10°, magnification of up to 1.1, lighting variations and warping. We further utilised the mix-up method during training, originally introduced by Zhang et al. [18], in which images of different classes (nr-axSpA and r-axSpA) are combined during training to reduce memorisation of noisy labels and increase overall model robustness. As a loss function, we used cross entropy label smoothing, which reduced high-confidence predictions of the models, thus supporting regularisation and avoiding overfitting with subsequent improved generalisation of the models on new data (e.g., test dataset). The optimal learning rate for training was determined using a learning rate range test [19]. Model training was performed with cyclical [19], discriminative learning rates (as initially implemented by Howard and Ruder [20]) and a progressive re-sizing approach, starting with image sizes of 224 × 224 pixels (which is the default input size for the ImageNet pre-trained ResNet-50) and next increasing the resolution to 512 × 512 pixels and then to 768 × 768 pixels. During training, first only the last two classification layers of the model were trained, with the weights of the other network layers remaining frozen. A total of 100 epochs were trained, monitoring the area under the receiver operating characteristics curve (AUC) on the validation dataset and saving the model weights on every improvement. After 100 epochs, the weights of the model with the highest AUC value were re-loaded, the model was unfrozen and again trained for another 100 epochs (training all layers of the network), while monitoring the AUC and saving the weights at every improvement. This approach was repeated for all image resolutions. The size of the mini batches was 64 for 224 × 224 pixels, 32 for 512 × 512 pixels and 84 for 768 × 768 pixels. The training for lower resolutions could be performed at our local workstation, while for 768 × 768 pixels, computation has been performed on the HPC for Research cluster of the Berlin Institute of Health. Overall, model training took approximately 24 h on our local machine and an additional 6 h on the cluster. After training, Gradient-weighted Class Activation Mapping (Grad-CAM) was used to create activation maps for verification that the model actually used the sacroiliac joints to determine if definite radiographic sacroiliitis was present [21].

### Statistical analysis

Statistical analysis was performed using the “*R*” statistical environment (version 3.6), the “tidyverse”, “ROCR” and “irr” libraries [22–25]. Raw predictions of the model on the validation dataset as well as on the test dataset using an image resolution of 768 × 768 pixels were exported from the python environment as comma-separated values and imported into “*R*”. ROC curves and precision-recall curves were plotted, and the AUC was calculated. Three different cut-offs were chosen through repeated cross validation for the calculation of sensitivity and specificity, the first cut-off favouring sensitivity, the second favouring specificity and the third aiming at balancing both. Confusion matrices were constructed using the predefined cut-offs. Cohen’s kappa and the percentage absolute agreement were used to assess the agreement between the human readers and the network. Ninety-five per cent confidence intervals for calculated kappa values were estimated using bootstrapping with 1000 repetitions. A *p*-value of < 0.05 was considered statistically significant.

### Ethics approval

Both PROOF and GESPIC were approved by the local ethics committees of each study centre in accordance with the local laws and regulations and were conducted in accordance with the Declaration of Helsinki and Good Clinical Practice. The institutional review board of the Charité — Universitätsmedizin Berlin additionally approved GESPIC. Written informed consent was obtained from all patients.

## Results

Definite radiographic sacroiliitis in the opinion of two readers was present in 873 (65.9%) patients from the training set (PROOF, *n* = 1324) and in 150 (65.5%) patients from the validation set (PROOF, *n* = 229). In a total of 369 (27.9%) and 63 (27.5%) patients in the training and validation sets, respectively, there was a discrepancy between the local reader and central reader 1, which automatically resulted in the involvement of central reader 2. A total of 146 (11.0%) and 37 (16.2%) patients in the training and validation sets were re-classified after the central reading, meaning that, in these cases, the ratings of both central readers differed from the rating of the local reader.

In the test set (GESPIC), both readers agreed on the presence of radiographic sacroiliitis in 223 (48.7%) cases and on the absence of radiographic sacroiliitis in 129 (28.2%) cases and disagreed in 106 (23.1%) cases.

### Model performance in the validation dataset

**Fig. 2:**
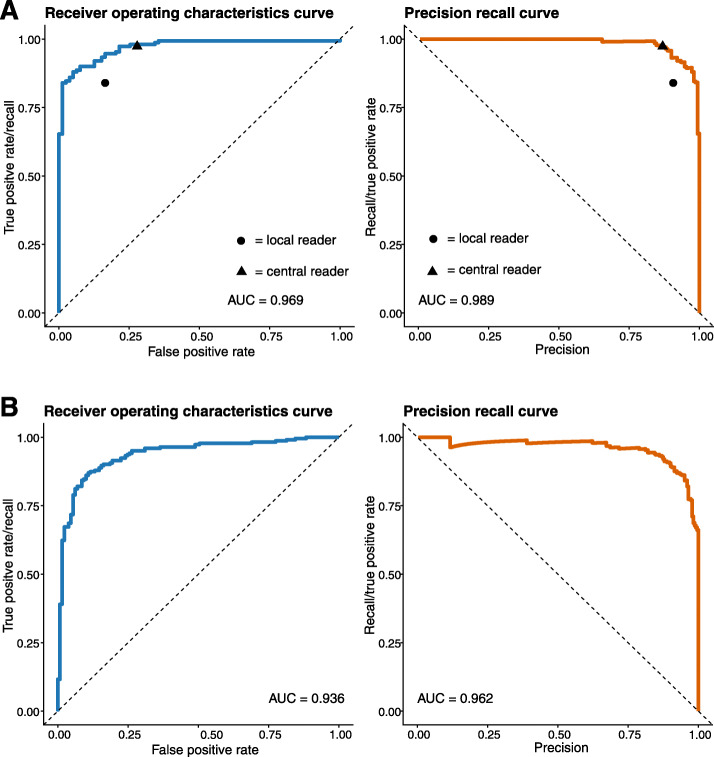
Receiver operation characteristics curve and precision-recall curve for the model performance in detecting definite radiographic sacroiliitis (classified as non-radiographic or radiographic axial spondyloarthritis) on the validation (**a**) and test (**b**) dataset as well as the corresponding area under the curve and average precision. Individual values for the local and the central expert reader are displayed as a triangle or dot in **a**. Since the reference standard in **b** was the agreement of two independent readers, their accuracy is not presented

There was excellent performance of the model on the validation dataset. The receiver operating characteristics curve (ROC) analysis showed an AUC of 0.969. For the precision-recall (PR) curve, an average AUC of 0.989 was achieved. Both the local and central expert readers remained below the ROC and PR curves and were therefore outperformed by the accuracy of the model. We propose three cut-offs to convert the floating-point predictions into integer values with 1 representing the presence of definite radiographic sacroiliitis and 0 its absence. Cut-offs weighting sensitivity over specificity and specificity over sensitivity were used in order to find the optimal balance between both parameters (defined as the maximum sum between sensitivity and specificity). The first cut-off value, which favours sensitivity over specificity, was calculated to be 0.475, resulting in a sensitivity of 0.993 and a specificity of 0.177 for the detection of r-axSpA. The second cut-off, which favoured specificity over sensitivity, was 0.787, resulting in a sensitivity of 0.753 and a specificity of 0.987. The third cut-off was 0.724, resulting in a sensitivity of 0.880 and a specificity of 0.949. ROC curves and precision-recall curves of model performance are shown in Fig. 2a, and Table 2 summarises performance results as confusion matrices with kappa values and values of absolute agreement.

**Table 2:** Confusion matrices for the three proposed cut-offs for the model predictions regarding the presence of definite radiographic sacroiliitis on the validation dataset

	nr-axSpA	r-axSpA	
Cut-off 1, favouring sensitivity over specificity			
Model predicts nr-axSpA	15	1	16
Model predicts r-axSpA	64	149	227
	79	150	229
Cohen’s kappa	0.22 (95% CI 0.11–0.33)	Accuracy:	*n* = 164/229 (71.6%)
Cut-off 2, favouring specificity over sensitivity			
Model predicts nr-axSpA	78	38	116
Model predicts r-axSpA	1	112	113
	79	150	229
Cohen’s kappa	0.66 (95% CI 0.57–0.76)	Accuracy:	*n* = 190/229 (83.0%)
Cut-off 3, optimal relationship between sensitivity and specificity			
Model predicts nr-axSpA	75	19	94
Model predicts r-axSpA	4	131	135
	79	150	229
Cohen’s kappa	0.79 (95% CI 0.7–0.87)	Accuracy:	*n* = 206/229 (90.0%)

### Model performance on the independent dataset

**Fig. 3:**
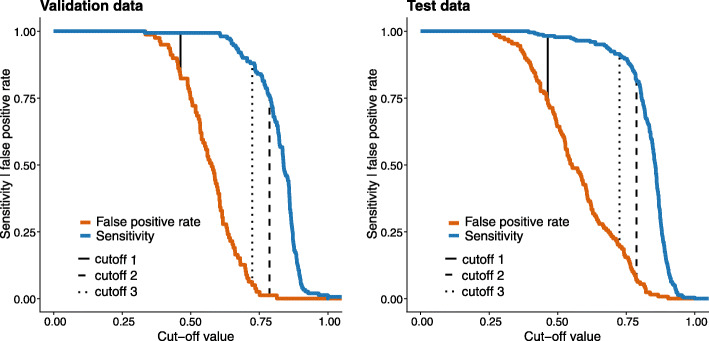
Sensitivity and 1-specificity (false positive rate) on the test and validation datasets using different cut-off values for the model predictions regarding the presence of definite radiographic sacroiliitis (classification as non-radiographic or radiographic axial spondyloarthritis). We analysed three cut-off values, indicated by vertical dashed lines. Cut-off 1 weights sensitivity over specificity, cut-off 2 weights specificity over sensitivity and cut-off 3 aims to be the optimal balance between the two performance measures. Cut-offs were only calculated on the validation dataset and then applied to the test and validation datasets

**Fig. 4:**
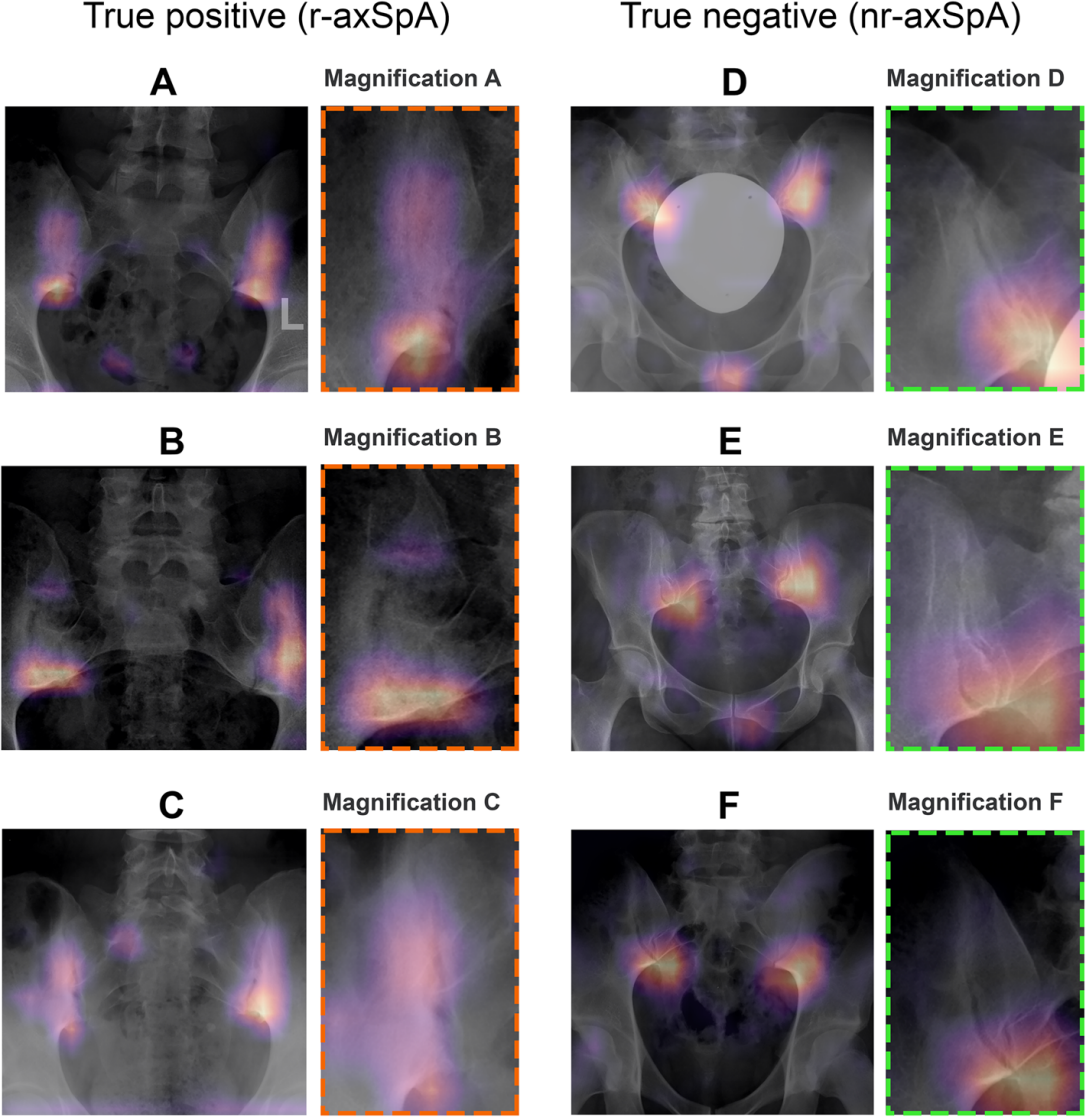
Gradient-weighted Class Activation Maps (Grad-CAMs) of the model for selected images taken from the test dataset. Grad-CAMs provide visual explanations for the model decision, as they highlight the important image regions that led to the model’s decision. The examples presented show that the model almost exclusively and correctly focusses on the sacroiliac joints to predict whether definite radiographic sacroiliitis is present or not

The model’s performance on the test dataset was assessed in two subsets. In the first subset, which comprised the cases where the two readers agreed on either the presence or absence of definite radiographic sacroiliitis (*n* = 352), the model performed slightly worse than on the validation dataset with an AUC value of 0.936 and an average precision (AP) value of 0.962. Again, we applied the three cut-offs as calculated from the validation dataset: The first cut-off, which weights sensitivity over specificity, yielded a sensitivity of 0.982 and a specificity of 0.264. For the second cut-off, which weights specificity over sensitivity, a sensitivity of 0.816 and a specificity of 0.930 were achieved. For the third cut-off, aiming at defining optimal performance in terms of both performance measures, we calculated a sensitivity of 0.915 and specificity of 0.806. Figure 2b shows the ROC- and precision-recall curves for the model performance on the test dataset. Figure 3 demonstrates the different values for sensitivity and specificity achieved for different cut-offs on the test and validation datasets. Table 3 provides confusion matrices for the three proposed cut-offs and the overall accuracy. Figure 4 shows examples of Grad-CAM maps of the neural network for predictions on the test datasets.

**Table 3:** Confusion matrices for the three proposed cut-offs for the model predictions regarding presence of definite radiographic sacroiliitis on the test dataset

	nr-axSpA	r-axSpA	
Cut-off 1, favouring sensitivity over specificity			
Model predicts nr-axSpA	36	4	40
Model predicts r-axSpA	93	219	312
	129	223	352
Cohen’s kappa	0.3 (95% CI 0.21–0.4)	Accuracy	*n* = 255/352 (72.4%)
Cut-off 2, favouring specificity over sensitivity			
Model predicts nr-axSpA	120	41	161
Model predicts r-axSpA	9	182	191
	129	223	352
Cohen’s kappa	0.7 (95% CI 0.63–0.77)	Accuracy	*n* = 302/352 (85.8%)
Cut-off 3, optimal relationship between sensitivity and specificity			
Model predicts nr-axSpA	104	19	123
Model predicts r-axSpA	25	204	229
	129	223	352
Cohen’s kappa	0.72 (95% CI 0.65–0.8)	Accuracy	*n* = 308/352 (87.5%)

For the second subset of cases, with disagreement between the two central readers (*n* = 106), the algorithm resulted in the following classification distribution: cut-off 1 (favouring sensitivity), 8 nr-axSpA and 98 r-axSpA; cut-off 2 (favouring specificity), 73 nr-axSpA and 33 r-axSpA; and cut-off 3 (balanced sensitivity and specificity), 53 nr-axSpA and 53 r-axSpA.

The interrater agreement between the human readers, as measured by Cohen’s kappa on the entire test dataset (*n* = 458), was moderate with *k* = 0.53 (95% CI 0.46–0.61) and a percentage agreement of 76.9%. The agreement between reader 1 and the neural network was similar with *k* = 0.54 (95% CI 0.46–0.62) and a percentage agreement of 77.3%. The agreement between reader 2 and the neural network was slightly better with *k* = 0.57 (95% CI 0.49–0.65) and a percentage agreement of 80.3%.

## Discussion

In this study, we successfully developed and tested an artificial intelligence model for the detection of radiographic sacroiliitis on conventional radiographs. With this model, we achieved an excellent model accuracy on the validation data. Furthermore, we demonstrated the generalisability of our model on a test dataset of novel data, achieving a performance at least comparable to that of two human experts.

Although magnetic resonance imaging is increasingly used for the detection of sacroiliitis in industrialised nations, radiographs are still important. In many countries, radiographs remain the first and often the only imaging procedure for examining patients with axSpA because MRI is expensive and not widely available. The detection of definite radiographic sacroiliitis is important for both the diagnosis and classification of axSpA. At the same time, it is well known that conventional radiographs are not very reliable in detecting sacroiliitis [6–10]. In the present study, we used a large and unique dataset to train, validate and test the model. The resulting performance was at least as good as (but most likely better than) the performance of an experienced reader with expertise in radiographic sacroiliitis assessment. The neural network was able to achieve almost the same level of performance in both the validation and training sets, indicating a high level of reliability and robustness of the model. Our model can therefore be used as an additional diagnostic aid in clinical practice and as a classification tool in research projects involving patients with axSpA.

Neural networks have already been applied to a variety of medical imaging data, including radiographs but, to our knowledge, not for the detection of spondyloarthritis [11–13, 26]. However, a low generalisability, i.e., poor performance of the models on new data, is an important challenge in training neural networks. A new meta-analysis on ‘deep learning performance against healthcare professionals’ by Kim et al. revealed methodological shortcomings that are present in many published studies on deep learning in medicine [27]. They criticised that many studies either did not compare the performance of their model with that of a human domain expert or assessed the performance of their model on a different dataset than the one used for human performance assessment, resulting in excessively high accuracies, mainly due to over-adaptation, which consequently have a low generalisability [27]. Similar observations were made by Yao et al., who showed that, while they identified 155 studies on deep learning in medicine, the studies often lacked external validation data [28]. However, the use of external validation data is an important measure to prove generalisability. It has been shown that medical computer vision models adapt poorly to the use of different scanners or imaging protocols, and the lack of external validation is likely to result in poor generalisability of the model to new data [29]. In a recent study, McKinney et al. evaluated the performance of a neural network for the detection of breast cancer in mammographs, showing that the network surpassed human performance [11]. They used different datasets from different studies to train and test their developed models and were thus able to demonstrate sufficient generalisability of their models.

Similar to their approach, we also used a heterogeneous training dataset with radiographs from different imaging sites and achieved a good generalisability of the developed model, with the performance on the test data being only slightly inferior. In our study, the test data were independent from the validation data in terms of both patients and readers [18, 30, 31]. While the heterogeneity of our training dataset already reduced the risk of overfitting on systematic image noise, e.g., to device-specific image features, we further increased generalisability by applying progressive re-sizing and the integration of mix-up as well as label smoothing into model training.

Our study has some limitations. First, the reference for the training of the model was the judgement of a limited number of human readers (2 or, in the case of discrepancy in the PROOF study, 3). Although both central readers in the PROOF study had many years of experience in the reading of radiographs of the sacroiliac joints, the complex sacroiliac joint anatomy and heterogeneity of radiographic techniques and quality have introduced some uncertainty into the final classification used as a reference. In the independent dataset, we selected primarily only cases where both readers agreed to be the reference standard for the evaluation of the model. This approach was chosen because we believe that these cases are most likely to be true positive or true negative, while in the cases with a discrepancy, the truth is not known. Nonetheless, the neural network-based classification of the discrepant cases was well balanced with the balanced cut-off indicating that our algorithm is also applicable in such rather difficult cases. It is noteworthy that, despite all the uncertainty related to the assessment of radiographic sacroiliitis, a high level of agreement between the neural network’s judgement and the human consensus judgement was achieved in both validation and test datasets.

Another limitation is related to the chosen sets — all patients were diagnosed with axSpA. The performance of the algorithm in patients with undiagnosed back pain and suspected axSpA in the diagnostic setting is not known and should be investigated in future studies.

## Conclusions

Radiographs of the sacroiliac joints are commonly used for the diagnosis and classification of axial spondyloarthritis, but the reliability of the definite radiographic sacroiliitis detection is usually low. Convolutional neural networks can detect radiographic sacroiliitis on pelvic radiographs with at least the same level of accuracy as a human expert. Utilisation of the proposed computer vision model could thus enable highly accurate detection of definite radiographic sacroiliitis, even in non-specialised sites.

## Data Availability

The data that support the findings of this study are available from the corresponding author, JLV, upon reasonable request.

## Abbreviations

AP: Average precision
AS: Ankylosing Spondylitis
ASAS: Assessment of Spondyloarthritis International Society
AUC: Area under the curve
axSpA: Axial spondyloarthritis
DICOM: Digital Imaging and Communications in Medicine
GESPIC: German Spondyloarthritis Inception Cohort
Grad-CAM: Gradient-weighted Class Activation Mapping
MRI: Magnetic resonance imaging
nr-axSpA: Mon-radiographic axial spondyloarthritis
PROOF: Patients With Axial Spondyloarthritis: Multicountry Registry of Clinical Characteristics
r-axSpA: Radiographic axial spondyloarthritis
ROC: Receiver operating characteristics curve
PR: Precision-recall
TIFF: Tagged Image File Format
